# Effect of group music therapy on state-anxiety and well-being levels of oncology patients undergoing chemotherapy: a multi-center randomized clinical trial

**DOI:** 10.3389/fpsyt.2025.1658503

**Published:** 2025-09-05

**Authors:** Mark Ettenberger, Laura Reyes-Aragón, Ana María Díaz, Raúl Suárez, Moshé Amarillo, Mariana Naranjo, Claudia C. Colmenares-Mejía

**Affiliations:** ^1^ Music Therapy Service, Clínica Sebastián de Belalcázar, Clínica Colsanitas, Cali, Colombia; ^2^ Music Therapy Service, Clínica El Carmen, Clínica Colsanitas, Barranquilla, Colombia; ^3^ SONO – Centro de Musicoterapia, Bogotá, Colombia; ^4^ Fundación Universitaria Sanitas, Research Group Investigación en patología clínica (INPAC), Grupo Keralty, Bogotá, Colombia

**Keywords:** music therapy, chemotherapy, anxiety, well-being, mental health, oncology

## Abstract

**Background:**

Cancer patients undergoing chemotherapy may experience several mental health challenges, including increased levels of anxiety and affected well-being. Music therapy and other music-based interventions have previously been applied to improve patient mental health during chemotherapy, but multi-site RCTs that report live group music therapy interventions are scarce.

**Methods:**

This is a multi-site randomized clinical trial (RCT) with two arms: a single live group music therapy session + standard care and standard care alone. Primary outcome measure was the State-Trait Anxiety Inventory (STAI), and secondary outcome measures were the Well-being Numerical Rating Scales (WB-NRSs). Between-group differences in STAI and WB-NRSs scores were analyzed using the Wilcoxon rank-sum test. Intra-individual pre-post changes were assessed with the Wilcoxon signed-rank test. This study follows the CONSORT guidelines for reporting RCTs.

**Results:**

A total of 110 patients were included in this study. Results showed a statistically significant reduction of state anxiety from pre- to post-intervention for the music therapy group (p<0.001), but not for the control group. Between-group analysis showed significantly lower post-intervention STAI scores in the music therapy group as compared to the control group (p<0.001). With respect to well-being, only the music therapy group had statistically significant increases in all dimensions of well-being within-group from pre-to post timepoints, and between-group analysis showed statistically significant post-intervention differences in psychological (p = 0.005) and general well-being (p = 0.030) favoring the music therapy group. Effects of hospital sites on the outcomes were not significant.

**Discussion:**

The results suggest that group music therapy during chemotherapy is a safe and effective strategy to improve mental health and well-being in cancer patients. To our knowledge, this is the first multicenter RCT on group music therapy during chemotherapy in Colombia. Future studies should aim at integrating caregivers of chemotherapy patients and seek expansion to an international multi-site RCT.

## 1 Background

Since chemotherapy treatments began in the early 1900s (1), it has remained one of the most widely used treatment options (2, 3), aiming to counteract the 20 million annual oncology diagnoses worldwide. With approximately 10 million victims every year, cancer is currently the second most common cause of death worldwide, a number expected to rise considerably in the coming years due to population growth, lifestyle, genetics, and diet (4). By 2050, a 77% increase of cancer cases is expected, reaching 35 million cases across the globe (5). In Colombia, 118,000 new cancer patients were diagnosed in 2022, a number projected to nearly double by 2045 (4, 5).

However, despite chemotherapy demonstrating effectiveness for specific types of cancer, it often causes significant physical, psychological, social, and spiritual burden for patients (3, 6). Several studies have shown that chemotherapy treatment is associated with increased levels of pain, anxiety and depression, as well as with greater emotional vulnerability, loss of identity, hopelessness, and diminished self-worth across different aspects of life (6–8).

About 60–70% of oncology patients receiving chemotherapy experience physical symptoms such as nausea, vomiting, alopecia, and pain (9–12). Additionally, neurological symptoms might also occur, such as neuropathy, difficulty in multitasking, deterioration of executive functions, impaired fine motor skills, or difficulty focusing and maintaining attention, collectively known as ‘chemo brain’ effects (12, 13).

Psycho-emotional states of cancer patients are affected by multiple factors, including information about the illness and treatment side effects (14). Studies indicate that 20–50% of chemotherapy patients experience mental health symptoms and require professional support to manage anxiety, stress, and depression associated with the disease (15). Particularly high anxiety levels are associated with more severe physical symptoms such as nausea and vomiting before and after chemotherapy treatment, impacting directly patients’ quality of life (9, 16). Additionally, secondary factors such as long waiting times and the noise of monitors and infusion pumps can increase psychological distress during chemotherapy (17). Such stressors can not only affect patients’ mental health and well-being, but also impact treatment adherence and tolerance, as well as disease progression and recovery (18).

To attenuate mental health challenges and chemotherapy-induced side effects for patients, various complementary therapies are being studied, including music therapy and other music-based interventions (19–21). Several meta-analyses highlight music’s potential to improve mental health and quality of life of cancer patients (22–25). With respect to chemotherapy, two recent meta-analyses report statistically significant improvements of music-based interventions and therapies for treatment-induced side effects such as vomiting and nausea (10) and patients’ anxiety levels and quality of life (26). However, the latter meta-analysis concluded that studies had a high risk of bias, and the quality of the evidence was rated from low to very low, particularly due to methodological concerns. Most individual studies conducted during chemotherapy sessions used pre-recorded music applied by other healthcare professionals than music therapists (27–30), and studies inclusive of music therapists mainly worked individually with patients (6, 31, 32). Only a few publications on group-based music therapy approaches during chemotherapy were found that provide some evidence for supporting emotional and mental health difficulties of patients in this setting A recent retrospective cohort study including 141 patients, and 51 caregivers highlighted the potential of group music therapy to reduce improve anxiety, stress, and well-being in patients and caregivers, and chemotherapy-induced side effects in patients (33). An in a prospective randomized study, Romito et al. (34) used a mix of vocal, improvisational, and non-musical elements (journaling, picture choice, etc.) with breast cancer patients and found significant reductions in stress, anger, depression, and anxiety. However, to our best knowledge, no previous multi-site studies on group music therapy has been conducted in this context.

The aim of this study is to determine the effect of a single live group music therapy intervention on state-anxiety and well-being levels in adult oncology patients undergoing chemotherapy at the hospitals Clínica Sebastián del Belalcázar (Cali, Colombia) and Clínica El Carmen (Barranquilla, Colombia).

## 2 Materials and methods

### 2.1 Study design

This was a pragmatic, multi-center, randomized clinical trial with two parallel arms: standard care + a single live group music therapy intervention during chemotherapy, and standard care alone during chemotherapy. This study follows the CONSORT guidelines for reporting randomized trials (35), and the CONSORT extensions for social and psychological interventions (36) and non-pharmacological treatments (37). Data collection lasted from October 21st, 2024, to March 13th, 2025.

### 2.2 Participants and setting

Participants were cancer patients undergoing outpatient chemotherapy at the hospitals Clínica Sebastián del Belalcázar and Clínica El Carmen. While located in different cities, both hospitals belong to the same healthcare provider with similar healthcare team structures, chemotherapy procedures, and care philosophies. The chemotherapy wards host from seven to ten patients and offer between two and four chemotherapy cycles per day. Medical and nursing staff are continuously present at the ward to aid with any issues that might arise during treatment.

### 2.3 Inclusion and exclusion criteria

The inclusion criteria were: adult patients over 18 years of age; patients with an oncology diagnosis and all types of cancer; undergoing outpatient chemotherapy at one of the hospitals; not having received music therapy previously; possessing the capacities to read, understand, and fill out the questionnaires; having signed an informed consent. The exclusion criteria were: patients with self-reported hearing impairment.

### 2.4 Selection of participants and randomization

Participants were invited to take part in the study through convenience sampling based on their availability during scheduled intervention times. The sessions were conducted on the same weekdays and time slots at both hospitals, twice a week on Mondays and Fridays, between 8:30 and 10:00 am. Allocation of participant groups to either the music therapy or control condition, as well as to the pre-defined intervention days, was carried out using block randomization of multiples of four with a computer-generated sequence (Microsoft Excel 365, version 2502). This ensured a balanced distribution of the intervention and control groups across study days and hospitals. Groups were pre-defined according to natural patient flow, and randomization was applied at the group (days) level.

### 2.5 Study procedure, masking, and concealment

Members of the healthcare team other than the music therapists were responsible for participant recruitment and informed consent. If patients agreed to participate, written informed consent was obtained, and the first set of questionnaires were handed out. The result of the randomization was revealed after filling out the questionnaires. For this, a pre-defined randomization was consulted shortly before the interventions by each music therapist at each hospital. Then, patients continued with standard care in case of the control condition or participated in a single live music therapy group session in case of the intervention condition. Both conditions lasted for the same amount of time (60 minutes). At the end, the second set of questionnaires were again handed out and collected by a member of the healthcare team. Due to the nature of the setting and intervention, participants and music therapists were not masked. Pre- and post-intervention data collection was performed by masked outcome assessors, and data analysis was performed by masked members of the research team.

### 2.6 Intervention description

#### 2.6.1 Music therapy group

The following intervention description is based on the updated guidelines for reporting music in intervention studies (38). Group-based music therapy interventions have demonstrated the capacity to facilitate communication, self-expression, and creative engagement among patients. Evidence from prior studies further suggests that participation in such interventions may promote a heightened sense of cohesion, belonging, and interpersonal intimacy within the group, which may ultimately result in improved mental health outcomes.

Before starting the group sessions, all instruments were cleaned following a previously published cleaning and disinfection protocol (39). The musical instruments used were an acoustic guitar with nylon strings (Yamaha C-40), an ocean drum (a double-skinned frame drum wrapped in synthetic leather, over which metal pallets roll imitating the sound of waves), a Samafon (aluminum tubes in different sizes that horizontally hang on strings and that are struck with a soft mallet), small percussion instruments (egg-shakers made of plastic), and a portable wireless Bluetooth speaker (JBL Go 3).

Then, the music therapist at each hospital (masters level) entered the chemotherapy ward, greeted the patients, outlined the structure of the music therapy group, and asked about musical preferences and current mood states. All participants remained seated in their respective infusion chairs during the intervention. To begin, soft music was played back (“Music Therapy Deep Relaxation in F Major” by Exomus Meditation), accompanied by gentle stretches considering mobility restrictions of each participant. The group was then invited to focus on their breathing and body sensations, followed by some moments of silence. This was followed by an activity focusing on movement, coordination, and body awareness, in which the music therapist suggested a simple movement fitting an up-beat music (“Dela” by Johnny Clegg), which everyone was invited to imitate (e.g., stamping to the beat of the music, or moving the arms following the melodic lines of the music). Then, if a participant wanted to, he or she could suggest his/her own movement, which again everyone imitated. To increase attention and playfulness, a number was assigned to each movement, and after some time, participants were invited to recreate the movements by using only the number as reference as well as fitting every movement with the music. The main part of the group session consisted in singing patient-selected songs with the group, supported by the music therapist playing the guitar and if participants opted to, accompanied by the group using the other available instruments. Songs were based on the musical preferences expressed by the participants at the beginning of the group session, but patients could also suggest new songs. The most common music genres at both hospitals were Boleros, Gospel music (both Catholic and Christian), Vallenatos, Salsa, and 60’s and 70’s Hispanic ballads (examples of songs are provided as a **Supplementary File**). Participants could either choose to sing and/or participate using the instruments, or to just listen. After that, the group could verbally reflect on the meaning of the songs and the music or share memories and emotions the songs brought up. To end the group session, patients were asked again to focus on their body sensations and breathing, while the music therapist played improvised and entrained live music on the guitar (e.g., I-IV or I-V arpeggiated chord progressions in 4/4, slow tempo, accompanied by the sound of the ocean drum and sometimes by humming or wordless singing). After the music faded out, participants could share their experiences, and verbal feedback was provided.

While treatment fidelity was not formally assessed in this study, the music therapists received the same intervention training and regular feedback and supervision with the principal investigator was provided to ensure coherence of the intervention across hospital sites.

#### 2.6.2 Control group

The control group received standard care only, consisting of routine medical and nursing procedures depending on patients’ needs and medical conditions, guaranteeing their physical, emotional, and mental well-being. Patients sat on their infusion chairs and continued with whatever they were used to during chemotherapy treatment, for example reading, working, listening to music on their personal devices, or sleeping.

### 2.7 Outcome measures

#### 2.7.1 Primary outcome measure

In this study, the Spanish version of the state-anxiety form of the State-trait Anxiety Inventory (STAI-E) was used as the primary outcome (40) and was applied before and after each intervention. The STAI was developed by Spielberger et al. (41) and consists of two forms with 20 items each rated on a 4-point Likert Scale, one for state anxiety and the other for trait anxiety. Total scores of the state-anxiety form range from 20 to 80, with higher scores indicating higher state-anxiety levels. Clinical cut-off scores are usually reported as ≥40 (42). The state-anxiety form of the STAI is a common outcome measure in music therapy and other music-based intervention studies during chemotherapy (27–29, 43).

#### 2.7.2 Secondary outcome measure

Secondary outcomes in this study were the Well-Being Numerical Rating Scales (WB-NRSs), developed by Bonacchi et al. (44). The WB-NRSs consist of 5 subscales for physical, psychological, social, spiritual and general well-being. Each scale consists of horizontal lines, accompanied by numbers ranging from 1-10, for which higher scores indicate higher well-being. The WB-NRSs were applied before and after each intervention.

#### 2.7.3 Socio-demographic and medical data

Additionally, socio-demographic and medical data, such as oncological diagnosis, number of chemotherapies received, sex, age, etc. were extracted from the electronic medical history.

### 2.8 Sample size

The sample size was calculated using Stata (version 13.0), based on expected mean differences between groups in the post-intervention scores of the primary outcome measure (STAI). To assess the treatment effect between groups, a two-way repeated measures analysis of variance (ANOVA) was conducted, assuming a statistical power of 0.80 and a significance level of 0.05. The following formula was applied:


$$z=\left[\left({\bar{x}}_{2}-{\bar{x}}_{1}\right)-\left(\mu_{2}-\mu_{1}\right)\right]/\surd \left[\left(\sigma_{1}^{2}/n_{1}\right)+\left(\sigma_{2}^{2}/n_{2}\right)\right]$$

Considering a 5% refusal rate, based on music therapists’ previous experience with cancer patients undergoing chemotherapy, and an estimated 15% prevalence of mental health disorders in the general population, the target sample size was set at 102 patients.

### 2.9 Data analysis

Qualitative variables were described using absolute and relative frequencies. Quantitative variables were summarized using measures of central tendency and dispersion, according to their distribution. The normality of quantitative variables was assessed using the Shapiro–Wilk test. Baseline characteristics were compared between the intervention and control groups using the Chi^2^ test for qualitative variables and the Student’s t-test for normally distributed quantitative variables. Differences in STAI and WB-NRSs scores between groups (control vs. music therapy before the intervention, and control vs. music therapy after the intervention) were analyzed using the Wilcoxon rank-sum test for independent samples. Intra-individual changes in STAI and WB-NRSs scores before and after the intervention were assessed using the Wilcoxon signed-rank test for paired data. A p-value <0.05 was considered statistically significant. Finally, to estimate the effect of the recruitment site (Hospital) on STAI scores, both crude and adjusted linear regression models were fitted. Results were reported as regression coefficients with corresponding 95% confidence intervals (95% CI). However, once the data were collected, the distribution of the STAI scores violated the assumptions of normality (as confirmed by Shapiro-Wilk test and histogram inspection). Therefore, non-parametric tests were used in the analysis: the Wilcoxon signed-rank test for within-group comparisons and the Mann–Whitney U test for between-group comparisons, to ensure appropriate statistical inference based on data characteristics.

### 2.10 Ethics approval and informed consent

This study was approved by the Research Ethics Committee of the Fundación Universitaria Sanitas (CEIFUS 2439-24, approval date: August 12th, 2024). All participants signed a written informed consent. This study was registered in clinicaltrails.gov (NCT06577324, submission date: August 21st, 2024). The publication of the study protocol has been submitted before patient recruitment (45).

## 3 Results

### 3.1 Patient characteristics

In this study, a total of 129 participants were randomized to a total of 28 music therapy group interventions or 26 control conditions. 19 participants (10 in the intervention group and 9 in the control group) presented incomplete data in the outcome measures. Thus, a total of 110 patients were included for analysis, with 50 assigned to the control group and 60 to the music therapy group. The mean age of the 110 participants was 55.5 years (SD: 15.4), with no significant difference between groups (p = 0.331). The overall sample comprised 64.5% females and 35.4% males, with a higher proportion of women in the music therapy group (71.7%) compared to the control group (56.0%), although this difference was not statistically significant (p = 0.087). Regarding the recruitment site, 39.1% of participants were recruited in Hospital A and 60.9% in Hospital B, with similar distributions between groups (p = 0.318). The median support network score was 10 (IQR: 9–10) in the overall sample, with no significant group differences (p = 0.326).

**Figure 1:**
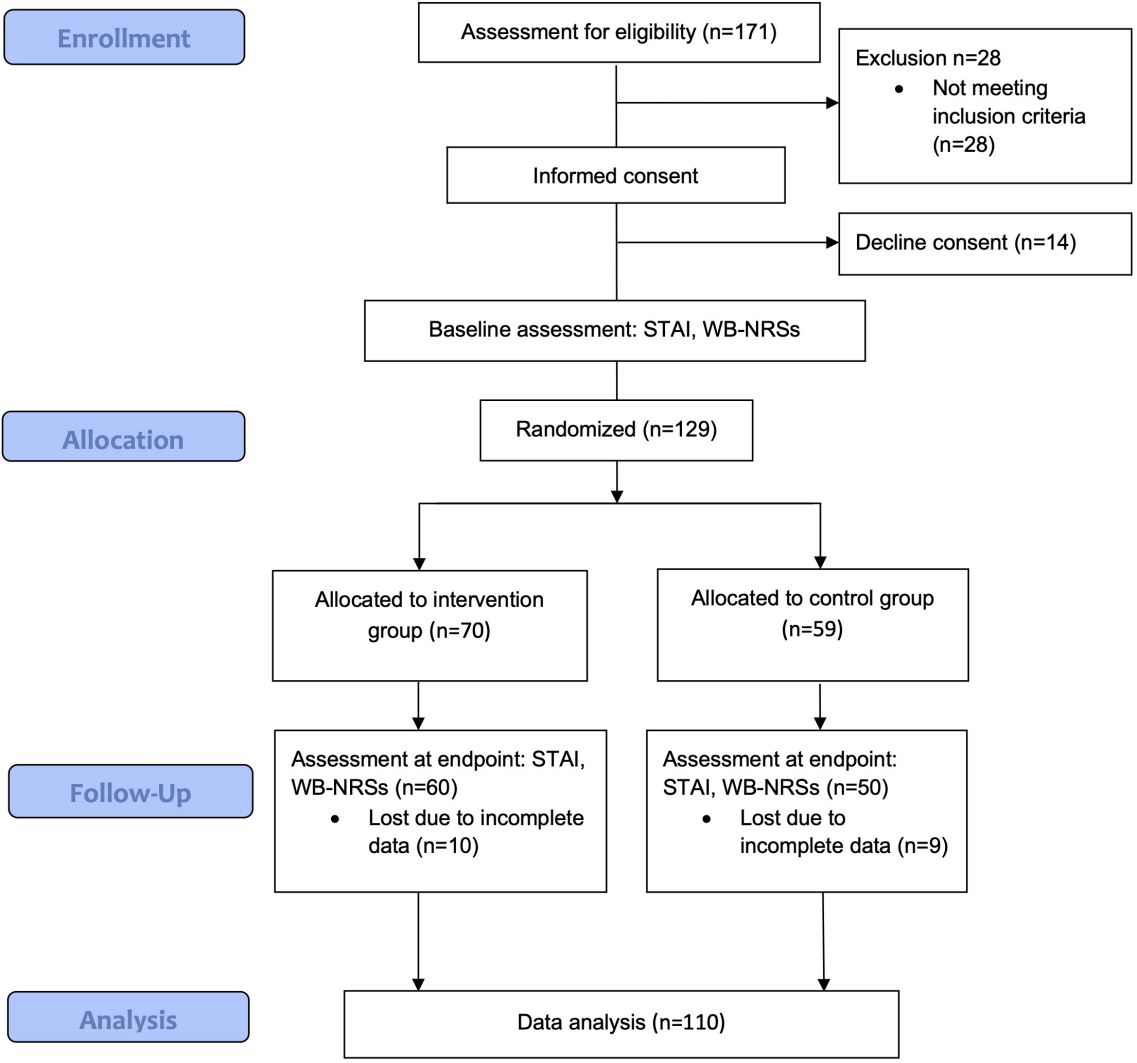
Flow-diagram.

Educational attainment varied: 31.8% of participants reported completing secondary education, and an equal proportion had vocational training. A small number held postgraduate degrees. Educational level distribution did not differ significantly between groups (p = 0.146). Regarding oncological diagnosis, the most common cancer type was breast cancer (37.3%), followed by hematolymphoid malignancies (12.7%), and colorectal cancer (10.9%). The distribution of cancer types was comparable between groups (p = 0.838). Most participants (82.7%) had received only one chemotherapy session in the current cycle, with no significant difference between the control (82.0%) and music therapy (83.3%) groups (p = 0.852). The number of previous chemotherapy cycles was also similar between groups (p = 0.779), with most patients having received one or two cycles. The patients medical and socio-demographic characteristics can be observed in **Table 1** and the flow-diagram of the study in **Figure 1**.

**Table 1:** Patients’ characteristics.

Variable	All N=110 N (%)	Control n=50 n (%)	Music Therapy n=60 n (%)	p
Age, years	55.5 (15.4)	57.1 (16.2)	54.2 (14.7)	0.331
Sex				0.087
Feminine	71 (64.5)	28 (56.0)	43 (71.7)	
Masculine	39 (35.4)	22 (44.0)	17 (28.3)	
City				0.318
Hospital A	43 (39.1)	17 (34.0)	26 (43.3)	
Hospital B	67 (60.9)	33 (66.0)	34 (56.7)	
Support network*	10 (9 - 10)	10 (10 - 10)	10 (9 - 10)	0.326
Education level				0.146
Primary education	12 (10.9)	8 (16.0)	4 (6.7)	
Secondary education	35 (31.8)	16 (32.0)	19 (31.7)	
Vocational education	35 (31.8)	17 (34.0)	18 (30.0)	
Undergraduate degree	21 (19.1)	5 (10.0)	16 (26.7)	
Master’s degree	5 (4.5)	3 (6.0)	2 (3.3)	
Doctoral degree	1 (0.9)	0 (0.0)	1 (1.7)	
Missing data	1 (0.9)	1 (2.0)	0 (0.0)	
Oncological diagnosis				0.838
Breast	41 (37.3)	15 (30.0)	26 (43.3)	
Hematolymphoid	14 (12.7)	5 (10.0)	9 (15.0)	
Colon and rectum	12 (10.9)	7 (14.0)	5 (8.3)	
Gastric	7 (6.4)	3 (6.0)	4 (6.6)	
Head and neck	5 (4.5)	4 (8.0)	1 (1.7)	
Ovary	5 (4.5)	3 (6.0)	4 (6.7)	
Lung	5 (4.5)	2 (4.0)	3 (5.0)	
Kidney	3 (2.7)	2 (4.0)	1 (1.7)	
Cervix	2 (1.8)	1 (2.0)	1 (1.7)	
Pancreas	2 (1.8)	1 (2.0)	1 (1.7)	
Prostate	2 (1.8)	1 (2.0)	1 (1.7)	
Thyroid	1 (0.9)	1 (2.0)	0 (0.0)	
Others	11 (10.0)	5 (10.0)	6 (10.0)	
Number of chemotherapy sessions received in the current cycle				0.852
1	91 (82.7)	41 (82.0)	50 (83.3)	
2	14 (12.7)	6 (12.0)	8 (13.3)	
3	5 (4.5)	3 (6.0)	2 (3.3)	
Received cycles of chemotherapy				0.779
1	32 (56.3)	27 (54.0)	35 (58.3)	
2	19 (17.2)	9 (18.0)	10 (16.6)	
3	13 (11.8)	6 (12.0)	7 (11.6)	
4	11 (10.0)	5 (10.0)	6 (10.0)	
5	2 (1.8)	2 (4.0)	0 (0.0)	
6	1 (0.9)	0 (0.0)	1 (1.6)	
8	1 (0.9)	0 (0.0)	1 (1.6)	
10	1 (0.9)	1 (2.0)	0 (0.0)	

*Median (IQR).

### 3.2 Main outcome measure: STAI

**Figure 2:**
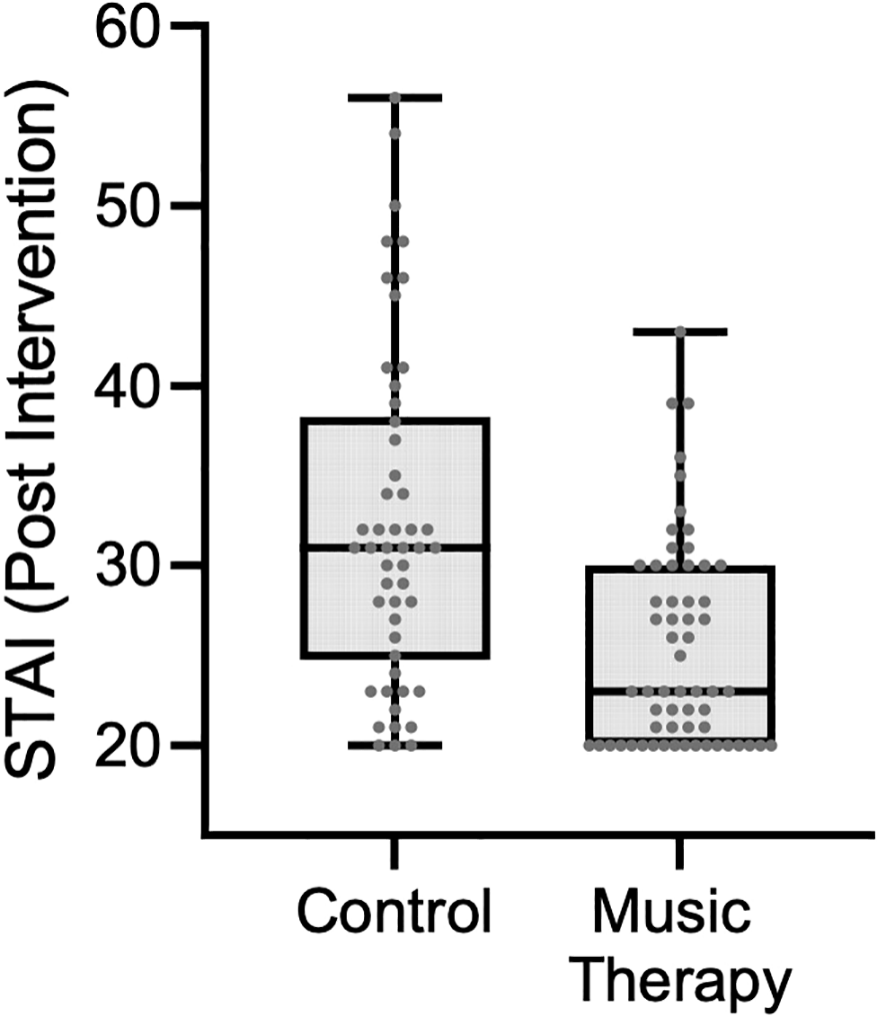
STAI scores by group post-intervention.

At baseline, there were no statistically significant differences in total STAI scores between the control group (median: 30.5; IQR: 27–37) and the music therapy group (median: 28; IQR: 24–37.5) (p = 0.271). Within the control group, STAI scores remained stable over time (pre: median 30.5, IQR: 27–37; post: median 31, IQR: 25–38; p = 0.726). In contrast, the music therapy group showed a statistically significant reduction in STAI scores following the intervention (pre: median 28, IQR: 24–37.5; post: median 23, IQR: 20–30; p < 0.001). Post-intervention, the music therapy group had significantly lower STAI scores compared to the control group (median: 23 vs. 31; p < 0.001), suggesting an anxiety-reducing effect of the intervention (**Figure 2**).

**Figure 3:**
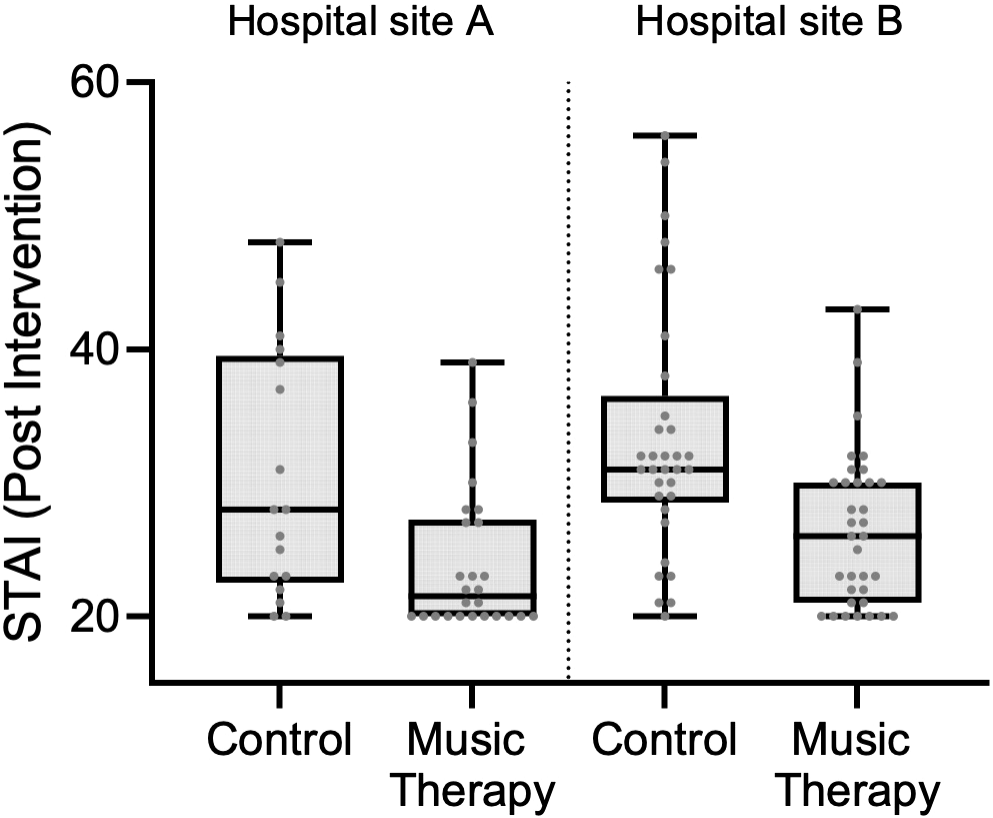
Post-intervention STAI scores by group and hospitals.

A stratified analysis was performed by healthcare institution. Among control group participants, post-intervention STAI scores were similar between the two hospitals: 28 (IQR: 23–39) in Hospital A and 31 (IQR: 29–35) in Hospital B (p = 0.185). In the music therapy group, post-intervention STAI scores were lower in Hospital A (median: 21.5; IQR: 20–27) compared to Hospital B (median: 26; IQR: 21–30), with a difference that approached statistical significance (p = 0.051) (**Figure 3**).

To evaluate the effect of recruitment sites on STAI scores, both unadjusted and adjusted linear regression models were constructed (**Table 2**). In the unadjusted model, participants in the music therapy group had significantly lower post-intervention STAI scores compared to those in the control group. Specifically, the music therapy group showed an average reduction of 7.03 points (95% CI: –9.91 to –4.15; p < 0.001). This model explained approximately 17.8% of the variance in STAI scores (R² = 0.1781), with a root mean squared error of 7.59, indicating that group assignment alone accounted for a modest but statistically significant portion of the variability in post-intervention anxiety levels. In the model adjusted for recruitment site, participation in the music therapy group remained significantly associated with lower STAI scores. On average, participants in the music therapy group had scores 6.78 points lower than those in the control group (95% CI: –9.64 to –3.92; p < 0.001). Additionally, participants recruited in Hospital B had STAI scores 2.68 points higher than those from Hospital A; however, this difference was not statistically significant (95% CI: –0.24 to 5.60; p = 0.072). The adjusted model explained approximately 20.3% of the variance in post-intervention STAI scores (R² = 0.2027), indicating a slightly improved fit.

**Table 2:** Crude and adjusted effects of music therapy on STAI scores.

Model	Size effect	SE	CI95%	p
Crude	-7.03	1.453	-9.91;-4.41	<0.001
Adjusted by recruitment site				<0.001
Hospital A	Ref	Ref	Ref	
Hospital B	-6.77	1.444	-9.64;-3.91	

#### 3.2.1 Subgroup analysis: STAI

**Figure 4:**
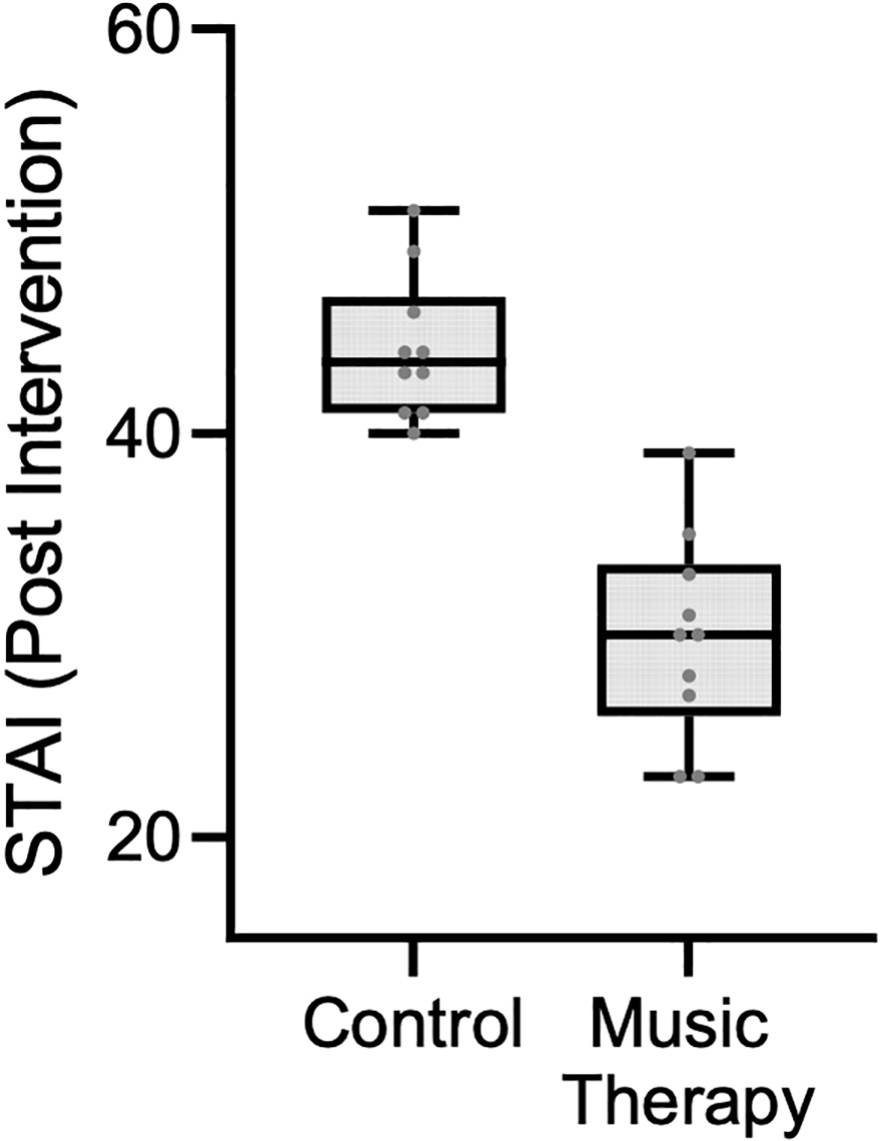
Post-intervention STAI scores among participants with baseline STAI ≥ 40 score.

A subgroup analysis was performed for 18 participants, who scored at baseline above the clinical cut-off for the STAI (≥40) (46). The median pre-intervention STAI score in the control group was 47.5 (IQR: 42.5–51.5), compared to 43.5 (IQR: 41–46) in the music therapy group, with no statistically significant difference (p = 0.180). Following the intervention, the median score was 47 (IQR: 43–51) in the control group and 30 (IQR: 27–33) in the music therapy group, showing a statistically significant difference (p < 0.001) (**Figure 4**).

### 3.3 Secondary outcome measure: WB-NRSs

At baseline, no significant differences were observed between the control and music therapy groups across any of the well-being dimensions: physical (p = 0.654), psychological (p = 0.543), social (p = 0.962), spiritual (p = 0.344), or general well-being (p = 0.782). After the intervention, the music therapy group showed a statistically significant improvement in the psychological domain (p = 0.005) and in the general well-being score (p = 0.030). Although there was a numerical increase in scores in the physical, social, and spiritual domains in the music therapy group, these changes did not reach statistical significance (p = 0.072, 0.117, and 0.096, respectively). In contrast, the control group exhibited no significant changes in any of the domains between the pre- and post-intervention assessments (**Table 3**).

**Table 3:** Comparison of between-group well-being scores (WB-NRSs) between control and music therapy groups pre- and post-intervention.

Wellbeing - NRS	Pre-intervention			Post-intervention		
	Control n=50 n (%)	Music Therapy n=60 n (%)	p	Control n=50 n (%)	Music Therapy n=60 n (%)	p
Physical	9 (7 - 10)	8.5 (7 - 10)	0.654	9 (7 - 10)	9 (8 - 10)	0.072
Psychological	9 (7 - 10)	9 (8 - 10)	0.543	9 (7 - 10)	10 (9 - 10)	0.005
Social	9 (8 - 10)	9 (8 - 10)	0.962	9 (8 - 10)	10 (9 - 10)	0.117
Spiritual	10 (9 - 10)	10 (9 - 10)	0.344	10 (9 - 10)	10 (9 - 10)	0.096
General	9 (8 - 10)	9 (8 - 10)	0.782	9 (8 - 10)	10 (9 - 10)	0.030

Within the control group, no statistically significant changes were observed across any of the well-being dimensions when comparing pre- and post-intervention scores: physical (p = 0.258), psychological (p = 0.455), social (p = 0.220), spiritual (p = 0.531), and general well-being (p = 0.868). These findings suggest stability in perceived well-being over time in the absence of the intervention. In contrast, the music therapy group demonstrated statistically significant improvements in all dimensions of well-being after the intervention. Physical well-being increased from a median of 8.5 (IQR: 7–10) to 9 (IQR: 8–10) (p < 0.001), psychological from 9 (IQR: 8–10) to 10 (IQR: 9–10) (p < 0.001), social from 9 (IQR: 8–10) to 10 (IQR: 9–10) (p < 0.001), and spiritual well-being, although already high, showed a modest but statistically significant increase (p = 0.021). Overall well-being also improved significantly from a median of 9 (IQR: 8–10) to 10 (IQR: 9–10) (p < 0.001) (**Table 3**).

## 4 Discussion

In this study, a statistically significant reduction in state-anxiety levels after a single group music therapy session in patients attending outpatient chemotherapy treatment was observed. Secondary analysis showed that patients with clinically significant levels of anxiety (STAI scores ≥40) scored below the cut-off after the intervention group condition (pre: 43.5 vs. post: 30.0), but not in the control group (pre: 47.5 vs. post: 47.0). Minimum clinically significant difference of the STAI has been reported to be 10 points (47, 48), meaning that the anxiety reduction was likely also clinically meaningful. With respect to well-being levels, statistically significant between-group differences were found for psychological and general well-being.

These findings are highly relevant, as many cancer patients experience mental health challenges during chemotherapy (15, 16). For breast cancer patients for example (37.3% of our study population), recent meta-analyses report a pooled prevalence of psychological distress of 50-52% (49, 50). For anxiety in particular, a prevalence of 20-31% has been found (51). Thus, investigating non-pharmacological and complementary strategies to improve mental health in this population is paramount, as high anxiety and depression levels are not only associated with worsened treatment-induced side effects (9), but also with cancer incidence, cancer-specific mortality, and all-cause mortality in cancer patients (52). However, overall anxiety levels of participants at baseline were relatively low. Thus, clinical relevance of the anxiety reduction can more easily be affirmed for the sub-group of participants with STAI scores ≥40, but for the general study population this remains inconclusive. As the median pre-intervention score across participants in the music therapy group was 28, but the minimum score of the STAI is 20, reaching the suggested minimum clinically significant difference of 10 points (47, 48) was not feasible.

While well-being is a common outcome measure during palliative care (53), to our best knowledge, it has not directly been measured in previous music therapy studies during chemotherapy. However, several studies report improvements in other resource-oriented outcomes such as sense of coherence and health locus of control (54), or quality of life (55), among others. Music therapy and other music-based interventions have a long history in oncology settings (21), but research is less common during chemotherapy treatment, and live group music therapy approaches are even more scarce. A recent retrospective cohort study showed improvements in anxiety, stress, and well-being levels of patients and caregivers after group music therapy during chemotherapy but used non-validated outcome measures (33). Romito et al. (34) provided a single integrative music therapy session for breast cancer patients during chemotherapy including music listening, group singing, and picture visualization for emotional expression. The results showed a statistically significant reduction in stress, anger, and anxiety for the music therapy group, but also a reduction of stress and anxiety for the active control group (one-to-one conversations with volunteers). Reduced depression, anxiety, helplessness, and cognitive avoidance levels were also found after group- vs. self-directed music interventions during chemotherapy by Chen et al. (56), but the groups were led by certified group therapists and the music was pre-recorded. A handful of studies used live music during chemotherapy, for example provided by orchestra musicians (29), or live Environmental Music Therapy (17), but without any active patient participation in music making. Thus, comparability of our results with other studies is limited and more evidence on group music therapy is needed before drawing further conclusions.

This is surprising, as from a clinical point of view, such groups can easily be implemented in chemotherapy wards and do not require major adjustments in terms of treatment schedule or help needed from the healthcare teams. Furthermore, in our study, no adverse events were reported and personal feedback from patients and staff was very positive. Considering the amount of time patients spend in chemotherapy, getting together in a group format also allows to strengthen interpersonal relationships among patients, and may over time help to establish a support group. Furthermore, such group sessions can play an important role in terms of fostering person-centered and humanized care in oncology and beyond (57). As cancer is one of the fastest growing diseases across the globe, creative, participative, and empowering arts- and music-based therapies and interventions focusing on mental health of patients during active treatment are needed.

### 4.1 Limitations

This study has several limitations. First, to our best knowledge, this is the first prospective trial on music therapy during chemotherapy in Colombia. Therefore, results should be interpreted with caution and generalizations to other contexts and settings are not feasible. Second, while both hospital sites belong to the same healthcare provider and share common features in terms of care offers and philosophies, more subtle cultural aspects, such as patients’ perceptions on music’s role for health, might have had an influence on the results. Third, due to the nature of the intervention, masking of participants and music therapists was not feasible. This is a common limitation in most music therapy studies (58). While all questionnaires were self-reported and data collection was done by a masked member of the research team, the lack of possibility to mask interventionists and patients might have resulted in bias. Fourth, we do not know if any effects regarding treatment-expectancy could have had an impact on the outcomes. This is an issue that has previously been mentioned in music therapy studies and options to measure treatment expectancy should be considered in future studies (59, 60). Fifth, we estimated 15% of participants with a previous mental health diagnosis as part of the sample size calculation for this study, but we were unable to confirm this estimation as this is not reported in the hospital system. However, as 16.3% of the participants (18 out of 110) scored above the STAI cut-off, it seems that at least for anxiety-related disorders, the estimation might have been correct. Also, it should be noted that there were slightly more participants in the intervention than in the control group. This is due to convenience sampling and the inclusion criteria of not having participated in previous music therapy experiences. Sixth, in general terms, anxiety levels across the study population were relatively low (means 30.5 for control 28.0 for music therapy) and well-being was high at baseline (median 9–10 for control and median 8.5-10). This means that most patients seemed to cope well during chemotherapy. This is different from previous studies that reported higher baseline anxiety levels (27, 28). While we can only speculate why our study population had low anxiety levels, in a future study, it would be important to focus on at-risk patients for mental health difficulties, particularly as the intervention effect seemed to be larger with participants scoring above the STAI cut-off. Seventh, about 18% (nineteen in total) of participants were excluded from the analysis due to incomplete responses in the psychometric scales, which precluded the calculation of total scores. As such, an intention-to-treat analysis could not be performed. This limitation should be addressed in future studies by improving data completeness and implementing strategies to enable ITT analyses. Eight, the difference in effect size between sites may reflect unmeasured contextual factors such as environmental conditions, organizational culture, or variations in patient engagement. However, these factors were not assessed in the present study and should be addressed in future research. Ninth, in this study caregivers were not invited to participate, as their presence at the chemotherapy wards is voluntary and fluctuating. As caregivers also experience elevated levels of stress and might benefit from music therapy, this should be considered in future studies (33). And lastly, in this study only a single group music therapy intervention was offered. This choice was made because we aimed for participants how had not previously been in music therapy before. However, we do not know if the treatment effect would have continued over time or if it would have increased or decreased across several sessions. Furthermore, no follow-up information is available on potential medium to long-term effects. In a future study, a process-oriented approach with several interventions could help answer such questions.

## 5 Conclusions

A single group music therapy session might be effective in reducing anxiety and improving well-being levels of patients during outpatient chemotherapy. This positions music therapists as important allies in the active treatment of oncology patients providing safe and effective interventions to improve mental health in this population. International multi-site studies are needed to confirm these preliminary results.

## Data Availability

The raw data supporting the conclusions of this article will be made available by the authors, without undue reservation.
