# Supplementary material for: Effect of group music therapy on state-anxiety and well-being levels of oncology patients undergoing chemotherapy: a multi-center randomized clinical trial
Source: Front Psychiatry. 2025 Sep 5;16:1658503. doi: 10.3389/fpsyt.2025.1658503 (PMC12447645; doi:10.3389/fpsyt.2025.1658503)
Supplement: Supplementary file 1 [file SupplementaryFile1.docx]

**Supplementary file: Songs used during the music therapy groups**

Boleros:

Piel canela - Bobby Capó

Sabor a mi - Álvaro Carrillo

Bésame mucho - Consuelo Velásquez

Contigo aprendí - Armando Manzanero

Cosas como tú - Armando Manzanero

Noches de Cartagena - Jaime Echevarría

Perfidia - Alberto Domínguez Borrás

El reloj - Lucho Gatica

Noches de Bocagrande- Trío Martino

Sin ti - Los Panchos

Somos novios- Armando Manzanero

Vallenato:

Las locuras mías - Silvestre Dangond

Mi hermano y yo - Hermanos Zuleta

Amarte más no pude - Diomedes Díaz

Tarde lo conocí - Patricia Teherán

Los caminos de la vida - Los diablitos

Mírame fijamente - Alejandro Durán

Alabanza (Gospel):

La Gloria de Dios - Ricardo Montaner y Evaluna

Al final - Lily Goodman

Al taller del maestro - Alex Campos

Ángeles de Dios - Marcelo Rossi

Sumérgeme - Jesús Adrián Romero

Levanto mis manos - Jesús Adrián Romero

El privilegio de amar - Mijares

Tú estás aquí - Jesús Adrián Romero y Marcela Góngora

Baladas:

Mi viejo - Piero

Amigo - Roberto Carlos

Un beso y una flor - Nino Bravo

La gata bajo la lluvia - Rocío Durcal

Ya te olvidé - Rocio Durcal

Un velero llamado libertad - José Luis Perales

¿Y cómo es él? - José Luis Perales

El amor de mi vida - Camilo Sesto

Chico de mi barrio - Tormenta

Llegando llegaste - Piero

Como te extraño - Leo Dan

Derroche - Ana Belén

Salsa:

Flor pálida - Polo Montañez

Plástico - Rubén Blades

Un montón de estrellas - Polo Montañez

En barranquilla me quedo - Joe Arroyo

Cali pachanguero - Grupo Niche

Vivir mi vida - Marc Anthony

Deseándote - Frankie Ruiz

Amor y control - Rubén Blades

Otros:

Color esperanza - Diego Torres

El pescador - José Barros

Aguacero de mayo - Toto la Momposina

Brindis - Thalía

Esta vida - Jorger Celedón

La gente luminosa - El Arrebato

Hoy puede ser un gran día - Joan Manuel Serrat

Éste es un nuevo día - Facundo Cabral
